# Reply to Learning by Doing….Errors! Comment on Gil-Lacruz, M.; Gracia-Pérez, M.L.; Gil-Lacruz, A.I. Learning by Doing and Training Satisfaction: An Evaluation by Health Care Professionals. *Int. J. Environ. Res. Public Health* 2019, *16*, 1397

**DOI:** 10.3390/ijerph17010038

**Published:** 2019-12-19

**Authors:** Marta Gil-Lacruz, María Luisa Gracia-Pérez, Ana Isabel Gil-Lacruz

**Affiliations:** 1 Health Science Faculty, University of Zaragoza, 50009 Zaragoza, Spain; 2 Social Sciences and Work Faculty, University of Zaragoza, 50009 Zaragoza, Spain; mlgracia@unizar.es; 3 School of Engineering and Architecture, University of Zaragoza, 50018 Zaragoza, Spain; anagil@unizar.es

We would like to thank the reviewer for the positive and constructive comments on our work and offer the following response:

Firstly, in accordance with the published literature, the learning that is acquired and applied during the training process very much depends on the level of implication of the student [1]. “Learning by Doing” is one of the training methodologies that requires the greatest implication of the participant. With reference to the specific programme, which was the basis for the article, the students themselves were of the opinion that the training methodology involved the highest degree of implication and was most useful in terms of applicability to their work in the organisation—of all training courses that they had undertaken.

The description of the FOCUSS programme indicates that the instructor is a professional, specialised in a specific technique that is taught to other professionals of related disciplines so they can use the knowledge and skills in their work. However, as our study focussed on the definition of the methodology it is possible that we have made assumptions on understanding and the practice of the methodology in the group of students: the instructor teaches, leads and evaluates a group of 3–6 students in the practice of a specific technique or skill. Once the students have completed the training they are able to take on the role of the instructor and pass on the new skills and knowledge to their workmates. 

Secondly, learning from mistakes and errors is something that every instructor incorporates in their management of the training group. In the FOCUSS programme, the average group size is four students, so the instructor is able to continuously supervise the training process and ensure that all mistakes are used as part of the learning process.

We would especially like to thank the reviewer for the comment referring to error management training as it offers us a new perspective for the future development and analysis of training programmes in which the registration of errors will undoubtedly be used as a definitive methodological tool.

## References

[1] Van Dam N., Fernández A., Porcel A.M., Nuviala A., Pérez R.J., Tamayo J., Grao A., González J.J. (2004). The e-Learning Fieldbook. Estudio Comparativo Entre Una Metodología de Aprendizaje Tradicional Respecto a Una Metodología de Aprendizaje Basada en el Learning by Doing Para la Consecución de Competencias Específicas. REVISTA Upo Innova.

